# Effect of E-glass fibers addition on compressive strength, flexural strength, hardness, and solubility of glass ionomer based cement

**DOI:** 10.1186/s12903-024-04447-8

**Published:** 2024-06-27

**Authors:** Tamer M. Hamdy

**Affiliations:** Restorative and Dental Materials Department, Oral and Dental Research Institute, National Research Centre (NRC), Giza, Dokki, 12622 Egypt

**Keywords:** Reinforcement, GIC, E-glass fibers, Compressive strength, Hardness, Solubility

## Abstract

**Background:**

In dentistry, glass-ionomer cements (GICs) are extensively used for a range of applications. The unique properties of GIC include fluoride ion release and recharge, chemical bonding to the tooth’s hard tissues, biocompatibility, a thermal expansion coefficient like that of enamel and dentin, and acceptable aesthetics. Their high solubility and poor mechanical qualities are among their limitations. E-glass fibers are generally utilized to reinforce the polymer matrix and are identified by their higher silica content.

**Objectives:**

The purpose of the study was to assess the impact of adding (10 wt% and 20 wt%) silane-treated E-glass fibers to traditional GIC on its mechanical properties (compressive strength, flexural strength, and surface hardness) and solubility.

**Methods:**

The characterization of the E-glass fiber fillers was achieved by XRF, SEM, and PSD. The specimens were prepared by adding the E-glass fiber fillers to the traditional GIC at 10% and 20% by weight, forming two innovative groups, and compared with the unmodified GIC (control group). The physical properties (film thickness and initial setting time) were examined to confirm operability after mixing. The evaluation of the reinforced GIC was performed by assessing the compressive strength, flexural strength, hardness, and solubility (*n* = 10 specimens per test). A one-way ANOVA and Tukey tests were performed for statistical analysis (*p* ≤ 0.05).

**Results:**

The traditional GIC showed the least compressive strength, flexural strength, hardness, and highest solubility. While the GIC reinforced with 20 wt% E-glass fibers showed the highest compressive strength, flexural strength, hardness, and least solubility. Meanwhile, GIC reinforced with 10 wt% showed intermediate results (*P* ≤ 0.05).

**Conclusion:**

Using 20 wt% E-glass fiber as a filler with the traditional GIC provides a strengthening effect and reduced solubility.

## Background

Polymers are commonly used in dentistry [1, 2]. The conservative dentistry procedures based on using adhesive restorative materials follow minimum tooth tissue removal while maintaining healthy tooth structure. Using restorative adhesive materials [3]. Glass ionomer cement (GIC) is a self-adhesive aesthetic restorative substance [4, 5]. Chemically, it is formed by an acid-base reaction. It is composed mainly of two components: weak polyacrylic acid and calcium fluoro-aluminosilicate glass fillers [6]. It has been used in many dental applications, including endodontic sealers, pit and fissure sealants, liner and bases, and minimally invasive and atraumatic direct restorative procedures [7].

GIC provides numerous benefits, including superior aesthetic qualities, fluoride release, chemical attachment to tooth structure, favorable thermal expansion, and biocompatibility [8, 9]. These materials have the ability to release and recharge fluoride over extended periods of time [10]. In the presence of calcium and phosphate ions, fluoride promotes the production of fluorapatite [10, 11]. When enough calcium and phosphate ions are present, it prevents the demineralization of enamel and dentin while encouraging remineralization at crystal surfaces. In early carious lesions, fluoride can prevent demineralization and encourage the remineralization of hard tooth tissues [11]. Furthermore, GIC has the capacity to release ions other than fluoride, such as calcium and aluminum; these ions promote the bioactivity and remineralization of enamel and dentin, mainly through the through the lactic acid buffering effect [12]. Even with these advantages, further improvement is required to overcome their drawbacks, which include their diminished mechanical properties and greater solubility rate. This could reduce their chances of longevity when they are used in regions that are subjected to high loads [13].Two critical physical attributes of a crucial component of restorative dentistry are film thickness and the dental cement’s initial setting time, which give an indication of the workability of the cement [13].

The development of new polymeric structures and the use of inorganic fillers led to advancements in dental materials [14]. Surface treatment, grafting, and the addition of reinforcing fillers are examples of filler modifications that are successful in enhancing dental materials and extending their life span [15–18].

The objective of many studies was to enhance the mechanical properties of the GIC. This was achieved by incorporating various filler particles, such as metallic fillers such as titanium, silver, graphene, and carbon [19, 20], or bioactive fillers such as hydroxyapatite particles and bioactive glass [18, 21–24].

The majority of masticatory forces in the posterior region of the oral cavity are compressive [25–27]. Therefore, the most crucial mechanical characteristic of restorative materials is compressive strength. Restorative materials with inferior compressive strength are more likely to break [25, 26]. Compressive strength may be regarded as a crucial success indicator, as restorative materials with enhanced compressive strength can withstand masticatory and parafunctional stresses [28]. A material’s flexural strength is a crucial characteristic that assesses the materials resistance to bending or fracturing under stresses [29]. Flexural strength is a suitable indicator of GIC strength since it reflects a clinical situation in which the restoration is being stressed by an opposing tooth [30]. It is an important aspect influencing how long any restoration lasts [31].

Nowadays, E-glass fiber reinforcement has emerged as an innovative and attractive approach in dentistry. It could provide an enhancement to the mechanical and physical qualities of dental materials by employing E-glass fibers in their composition. E-glass fibers have been the most frequently used fibers in dentistry due to their superior bonding with dental polymers and acceptable aesthetics [32].

A variety of variables, such as fiber diameter, length, orientation, percentage, and adhesion, affect the properties of fiber-reinforced materials [3]. Enhancing the mechanical qualities of the resulting structure requires improved adhesion between the fiber and matrix, which could be substantially improved by the silane treatment of the fiber [33, 34]. Therefore, the present study is intended to evaluate the impact of adding (10 wt% and 20 wt%) silane-treated E-glass fibers to traditional GIC on its compressive strength, surface hardness, and solubility and compared with the unmodified GIC (control group). The null hypothesis stated that the addition of E-glass fiber fillers to the traditional GIC at 10% and 20% by weight has no influence on the compressive strength, flexural strength, hardness, or solubility in comparison to the untreated control group.

## Methods

The present experimental study was approved by the Medical Research Ethical Committee (MREC) of the National Research Centre (NRC), Cairo, Egypt (reference number: 305,032,023). For this study, a commercially conventional chemically cured GIC was utilized in powder and liquid form: Fuji IX GP Extra (GC Corporation, Tokyo, Japan). Commercial E-glass fiber powder was used as a filler (Fibertec Inc., Scotland Boulevard, Bridgewater, MA, U.S.A.). The details of the materials used in the current study are listed in Table (1).

**Table 1:** The detailed data on the used materials in the study

Material	Manufacturer	Composition	Batch number	
Fuji IX GP Extra	GC Corporation, Tokyo, Japan.	Polyacrylic acid, fluoro-alumino-silicate glass, other ingredients.		002578
Microglass Milled Fiber 9110 Series	Fibertec Inc., Scotland Boulevard, Bridgewater, MA, U.S.A.	Silane treated E-glass fiber, consists of highly transparent high aspect ratio E-glass fiberglass, 16 μm diameter, 150 μm length. SiO_2_ (50–55 wt%), CaO (20–24 wt%), MgO (20–24 wt%), B_2_O_3_ (1–3 wt%), Al_2_ O_3_ (4- 6wt%).		030095

### Chemical and morphologic characterization of E-glass powder

#### X-ray fluorescence (XRF) analysis

The qualitative chemical analysis of the E-glass powder was carried out by non-destructive XRF analysis (X-MET3000TXR, Oxford Instruments GmbH Co., Borsigstrasse, Germany) to verify the chemical composition of the used fillers [35]. The instrument examination was performed at 40 kV, 40 mA, and 1600 W.

#### Scanning electron microscope (SEM)

The morphological analysis of the E-glass shape and distribution were examined via scanning electron microscopy (SEM) (Quanta 250 FEG, FEI Company, Hillsboro, OR, USA). It was carried out with an accelerating voltage range of 20.0 kV to 30.0 kV, and the magnification was 1600X. The E-glass fibers were examined before mixing as well as after mixing with the GIC.

#### Particle size distribution (PSD) analysis

The particle size of E-glass fiber fillers was examined using a particle size analyzer with a NICOMP 380 ZLS dynamic light scattering (DLS) instrument (PSS Nicomp 380 particle sizer, Santa Barbara, California, USA). The PSD of the E-glass particles was investigated. Based on histogram analysis, the average particle diameter of E-glass fiber particles was provided. The Gaussian particle size distributions and NICOMP distribution curves were determined.

#### Physical characterization of the mix

Film thickness and setting time measurements were used to evaluate the physical characteristics of the control and treated specimens. Following the guidelines of ISO standard 9917-2 from the International Standard Organization (ISO), the film thickness of the control and treated specimens was examined [36]. Four measurements were made of the thickness of two flat glass plates that were connected together, to the nearest 0.1 m, using an electronic digital caliper (Digital Vernier Calliper, Mitutoyo, Japan). A record of this reading was made (reading A). According to the manufacturer’s directions, the cement for each group (*n* = 10) was mixed. Following mixing, each cement mixture was equally divided between the two glass plates. The upper glass plate was subjected to a load of 147 N using a universal testing apparatus (Shimadzu Autograph AG-X Plus, Kyoto, Japan). After seven minutes, the total thickness of the plates with the specimen in between was noted as reading B. The difference between the thickness of the plates with and without the material between (B-A) was used to calculate the final total film thickness for the specimen being tested [36].

The Gillmore needles (Humboldt MFG., Norridge, IL, USA) were used to determine the initial setting times in accordance with ADA guidelines [37]. A light needle weighing 113.4 g and having a tip diameter of 2.12 mm was used to calculate the initial setup time. The needle was positioned on the surface every 30 s. The initial setting times were calculated starting from the point at which mixing began and ending when the needle left no surface marks, respectively. For every group, ten samples were measured [38].

#### Sample size calculation

The sample size calculation was based on a similar study [3, 30]. With an alpha level of 0.05 and a power of 85%, a sample size was determined using G*Power software version 3.1.9.7 (Heinrich Heine University Duesseldorf, Duesseldorf, Germany). The minimum sample size needed with this effect size is (*n* = 10 per group) to test compressive strength, flexural strength, microhardness, and solubility.

#### Study design

**Fig. 1:**
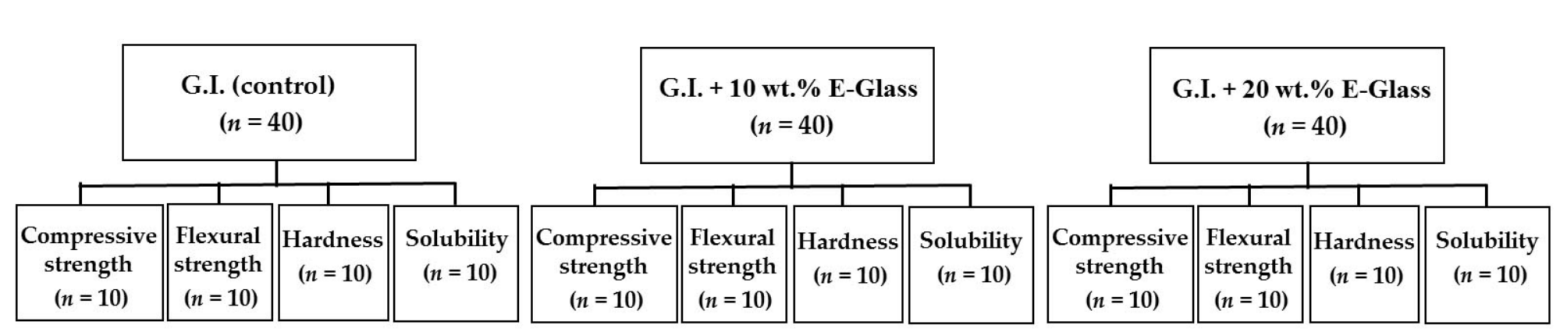
Distribution of groups according to materials and types of tests

A total of 120 specimens were prepared according to each type of the analytic test. They were standardized and evenly distributed in three groups of 40 specimens. These were divided into three subgroups of specimens (*n* = 10) for examination of compressive strength, flexural stregth, microhardness, and solubility, as represented in Fig. 1.

#### Sample preparation

The preparation of the control group was done by mixing the conventional GIC powder with their liquid. While the reinforced groups were obtained by mixing 10 wt% and 20 wt% E-glass fiber powder, respectively, with the conventional GIC powder, using an amalgamator, to reach a homogenous powder mixture. The prepared powder was then mixed with the GIC liquid. The mixing of powder and liquid was done according to the manufacturer’s instructions. The mixed material from each group was then filled into specially designed molds according to the test specifications.

### Analytic tests

#### Compressive strength test

In accordance with ISO standards 9917-2:2017 for water-based cements, compressive strength was assessed for each group [39]. Ten cylindrical specimens (*n* = 10) were created for each group using a split Teflon mold with internal dimensions (measuring 6 ± 0.1 mm in height and 4 ± 0.1 mm in diameter). The cement paste was loaded into the mold with a syringe. The top and bottom surfaces of the surface were covered with a celluloid strip and glass slide. The specimens were stored at 37 °C and a relative humidity of 100% for 60 min. and were carefully removed from the molds, then stored for 23 h at 37 °C in deionized water. The excess cement was eliminated by polishing both sides with 500-grit carbide paper under water irrigation on a grinder-polisher (Buehler, IL, USA) to reach the proper dimensions of 4 mm in diameter and 6 mm in height. Compressive strength tests were performed using a universal testing machine (Shimadzu Autograph AG-X plus 5 kN, Kyoto, Japan) with a crosshead speed of 1 mm/min. Specimens were loaded in compression until a fracture occurred. The compressive strength was determined in MPa using the following formula [39]:

Compression strength = $$\frac{4P}{\pi {d}^{2}}$$

Where: P is the fracture load (N); d is the diameter (mm).

#### Flexural strength test

In accordance with ISO 20795-1, a flexural strength test was assessed utilizing 3-point bending [40]. . Specimens measuring 64 mm in length, 10 mm in width, and 3.3 mm in thickness were created using a metallic mold [40]. . A universal testing machine (Model 3345; Instron Industrial Products, Norwood, MA, USA) was used to evaluate the specimens. Using a load cell of 500 N, the load was delivered to the center of the specimens, which were maintained over a 2-point support span of 50 mm apart and a crosshead speed of 5 mm/min. Until they broke, the specimens were loaded. In Newtons (N), the load at fracture was expressed. The following formula was used to compute the flexural strength (FS) in MPa [41, 42]. : FS = 3PL/2bh^2^. Where (L) is the distance between the supports (mm); (b) is the breadth (mm); (h) is the height of the specimen (mm); and (P) is the maximum load at fracture (N).

#### Vickers hardness (VH) test

Ten disc-shaped specimens (*n* = 10) per group measuring 5 mm in height and 2 mm in diameter were prepared using Teflon mold [3]. The hardened specimens were removed from the molds and subsequently immersed in distilled water and maintained at 37 °C for 24 h in a highly humid incubator. After 24 h, the specimens were removed from the solution and dried. Surface microhardness for each specimen was determined using a digital Vickers hardness tester (NEXUS 400TM, INNOVATEST, model no. 4503, Maastricht, Netherlands). The indentations were made within 10 s of dwell time at a load of 100 g at 40 x magnificence. The results were expressed in Vickers hardness numbers (VHN) automatically using the formula [3]:

VHN = 1.8544 P/d^2^, where (p) is the applied force in kilograms and (d) is the mean of the two diagonals gained from the indentation in mm.

Each specimen was indented three times and averaged to calculate the mean Vickers microhardness values (VHN).

#### Solubility percentage test

Solubility was tested using a Teflon mold that measured 7 mm in diameter and 2 mm in thickness, producing a disc-shaped specimen (*n* = 10) [43]. Every group’s specimens were kept for two hours in a desiccator filled with silica gel (Merck KGaA, Darmstadt, Germany), and then for a further twenty-two hours, they were incubated at 37 °C. First mass (M1) values were obtained by weighing specimens with an accuracy of 0.001 g on a precision analytical balance (Adam Equipment 4 digits precision weighing balance, Adam Equipment Inc., Oxford, UK). The samples were then kept for seven days and incubated at 37 °C for seven days after being submerged in a plastic flask filled with 25 mL of distilled water. To obtain the mass values of the specimens after immersion (M2), each specimen was then taken out and carefully dried with absorbent paper [44]. Using the equation, the percentage of solubility was determined [45]:

$$(M1-M2)/M1\,\times\,100\%$$

where M1 is the initial mass and M2 is the final mass of the specimens. The test was repeated three times [43].

#### Statistical analysis

The Statistical Package for the Social Sciences (SPSS) 16.0 statistical program (IBM-SPSS version 27.0, New York, NY, USA) was used to conduct the statistical study. Using the Kolmogrov-Smirnov and Shapiro-Wilk tests, the data revealed a normal distribution. Analysis of Variance (ANOVA) and Tukey tests were utilized to compare the mean film thickness (µm), initial setting time (seconds), compressive strength (MPa), flexural strength (MPa), hardness (VHN), and solubility (%) for the glass ionomer (control), G.I. reinforced with 10 wt% E-glass, and G.I. reinforced with 20 wt% E-glass. The significance level was set at *P* ≤ 0.05.

## Results

### XRF characterization results

The chemical composition of E-glass powder analyzed by XRF is shown in Table (2). The XRF results revealed that SiO_2_ constituted most of the fibers, which weighed about 53 wt%. MgO and CaO contents were 20 and 21 wt%, respectively. The Al_2_O_3_ concentration was 4 wt%. There was 1 wt% of K_2_O, and 1 wt% of B_2_O_3_.

**Table 2:** Chemical compositions (wt%) of E-glass determined by XRF analysis

Chemical composition	wt%
SiO_2_	53
Al_2_O_3_	4
B_2_O_3_	1
MgO	20
CaO	21
K_2_O	1

### SEM characterization results

**Fig. 2:**
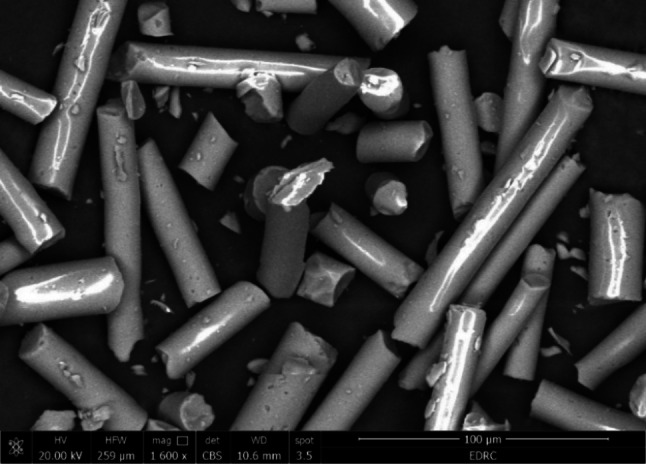
SEM image of E-glass fibers

As seen in Fig. 2, the SEM micrograph of the E-glass fibers was acquired at a magnification of 1600 X. The SEM scans showed a short, homogeneous, straight filament morphology. Furthermore, the fibers had a smooth and shiny surface and were uniformly dispersed.

**Fig. 3:**
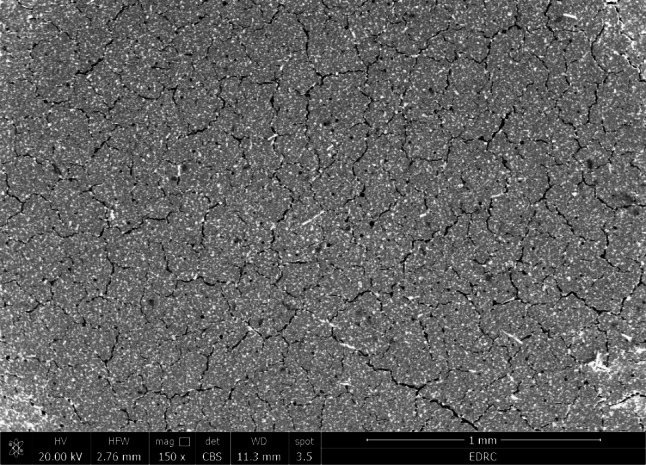
SEM image after mixing of 10 wt% E-glass fibers with GIC at 150 X magnification

**Fig. 4:**
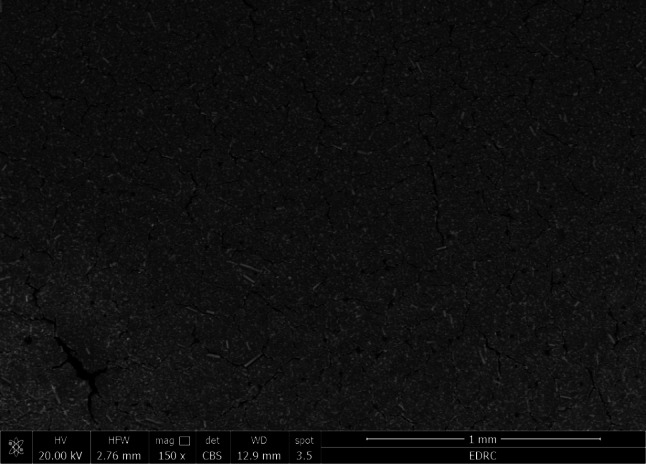
SEM image after mixing of 20 wt% E-glass fibers with GIC at 150 X magnification

**Fig. 5:**
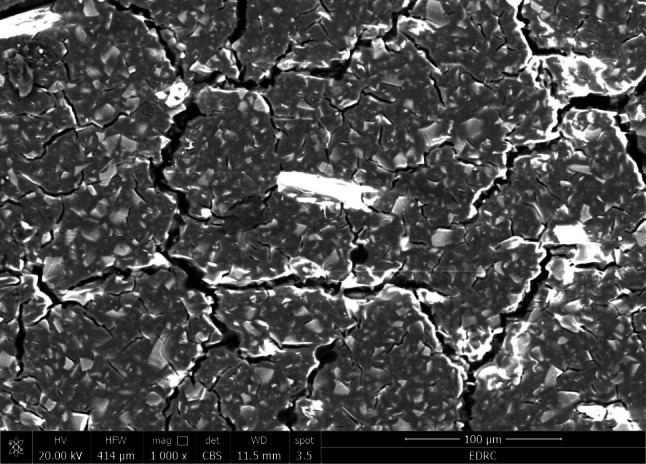
SEM image after mixing of 10 wt% E-glass fibers with GIC at 1000 X magnification

**Fig. 6:**
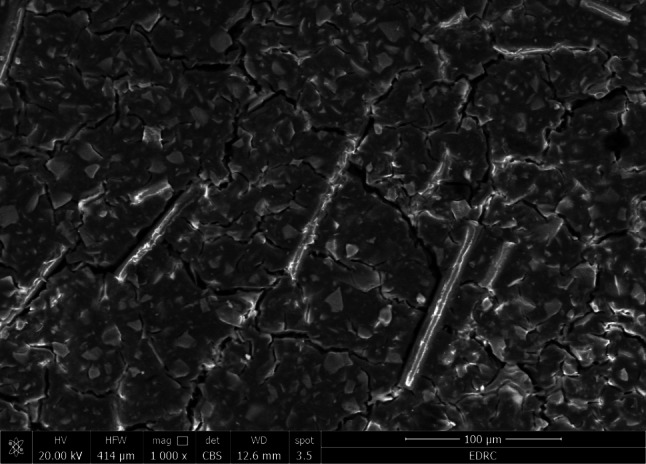
SEM image after mixing of 20 wt% E-glass fibers with GIC at 1000 X magnification

As shown in Figs. (3, 4, 5 and 6), the SEM micrograph of the E-glass fibers (10 wt% and 20 wt%) after mixing with GIC E-glass was acquired at a magnification of 150 X and 1000 X. The SEM scans showed a uniform distribution of the short glass fibers in both 10 wt% and 20 wt% concentrations. Moreover, the bonding between glass fibers and GIC appears to be tight. Furthermore, some cracks were exhibited on the surface of GIC.

### Particle size characterization results

**Fig. 7:**
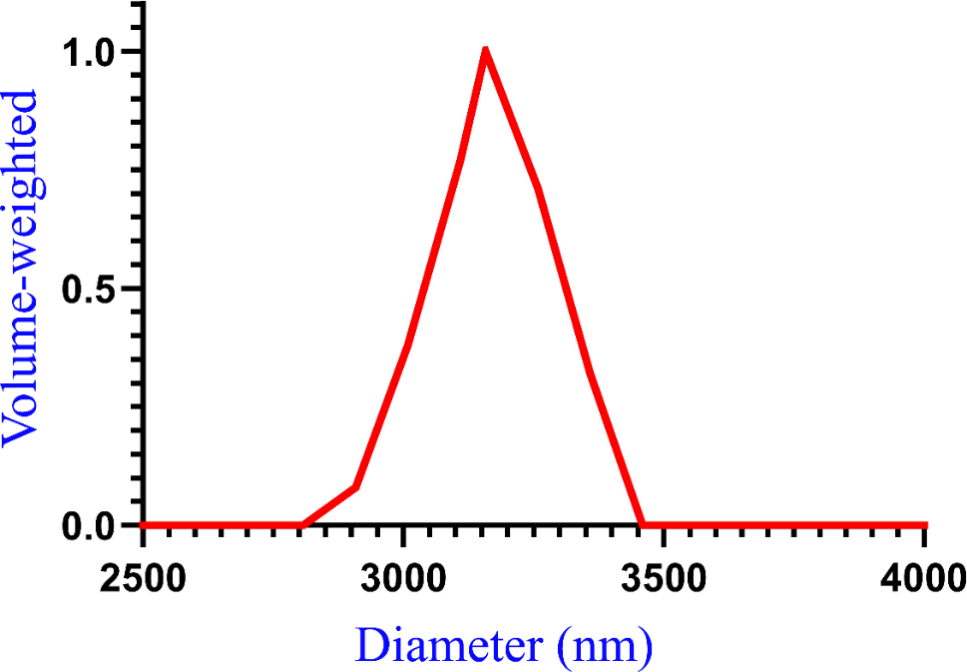
E-glass fibers diameter distribution

The diameter distribution of the E-glass fibers analyzed by DSL is plotted in Fig. 7. The diameter distribution of the E-glass fibers according to Gaussian distribution analysis can be summarized as follows: The intensity-weighted Gaussian distribution mean diameter was 1.1307 μm, and the volume-weighted Gaussian distribution mean diameter was 3.1579 μm. In addition, the mean length distribution was 315.79 μm. Moreover, the mean aspect ratio was 100.

### Physical characterization results

#### Film thickness

Table 3 displays the film thickness data for the control and reinforced specimens. There was no significant difference (*P* = 0.7) in the film thickness values between the untreated GIC (control) group (22.4 μm) and the GIC reinforced with 10 wt% and 20 wt% E-glass groups (23.4 μm and 23.8 μm, respectively).

**Table 3:** The film thickness mean and standard deviation values among the groups (µm)

Test	GIC (control)	GIC reinforced with 10 wt% E-glass		GIC reinforced with 20 wt% E-glass	*P* value
Film thickness (µm)	22.4 ± 1.1		23.4 ± 0.5	23.8 ± 0.8	0.7

#### Setting time

Table 4 shows the control and reinforced specimens’ initial setting time results. There was no significant difference (*P* = 0.1) in the initial setting time between the untreated GIC (control) group (98.8 s) and the GIC reinforced with 10 wt% and 20 wt% E-glass groups (100.6 and 100.8 s, respectively).

**Table 4:** The initial setting time mean and standard deviation values among the groups (seconds)

Test	GIC (control)	GIC reinforced with 10 wt% E-glass		GIC reinforced with 20 wt% E-glass	*P* value
initial setting time (seconds)	98.8 ± 1.9		100.6 ± 0.5	100.8 ± 1	0.1*

### Analytical test results

#### Compressive strength results

Table 5 presents the results of the compressive strength. The mean values of the three groups varied significantly from each other. The GIC (control) showed the least compressive strength (96 MPa), While the GIC reinforced with 20 wt% E-glass showed the highest compressive strength (136 MPa), Meanwhile, GIC reinforced with 10 wt% showed intermediate results (115.2 MPa), (*P* = 0.0001*).

**Table 5:** The compressive strength mean and standard deviation values among the groups (MPa)

Test	GIC (control)	GIC reinforced with 10 wt% E-glass		GIC reinforced with 20 wt% E-glass	*P* value
Compressive strength (MPa)	96^a^ ± 3.2		115.2^b^ ± 3.3	136^c^ ± 4	0.00001*

Different small letters in the same row are significant difference, * denotes significant difference as *P* ≤ 0.05

#### Flexural strength results

Table 6 shows the results of the flexural strength. The mean values of the three groups varied significantly from each other. The GIC (control) showed the least flexural strength (47 MPa), while the GIC reinforced with 20 wt% E-glass showed the highest flexural strength (76.4 MPa). Meanwhile, the GIC reinforced with 10 wt% showed intermediate results (57.2 MPa), (*P* = 0.0001*).

**Table 6:** The flexural strength mean and standard deviation values among the groups (MPa)

Test	GIC (control)	GIC reinforced with 10 wt% E-glass		GIC reinforced with 20 wt% E-glass	*P* value
Flexural strength (MPa)	47^a^ ± 1.6		57.2^b^ ± 1.3	67.4^c^ ± 1.1	0.0001*

Different small letters in the same row are significant difference, * denotes significant difference as *P* ≤ 0.05

#### Hardness results

Table 7 presents the results of the surface hardness. The mean values of the three groups varied significantly from each other. The GIC (control) showed the least hardness (44.4 VHN), While the GIC reinforced with 20 wt% E-glass showed the highest hardness (75.9 VHN), Meanwhile, GIC reinforced with 10 wt% showed intermediate results (58.8 VHN), (*P* = 0.0001*).

**Table 7:** The hardness mean and standard deviation values among the groups (VHN)

Test	GIC (control)	GIC reinforced with 10 wt% E-glass		GIC reinforced with 20 wt% E-glass	*P* value
Hardness (VHN)	44.4^a^ ± 0.6		58.8^b^ ± 1.4	75.9^c^ ± 3.5	0.00001*

Different small letters in the same row are significant difference, * denotes significant difference as *P* ≤ 0.05

#### Solubility results

Table 8 presents the results of the surface solubility. The mean values of the three groups varied significantly from each other. The GIC (control) showed the highest solubility (5.5%). While the GIC reinforced with 20 wt% E-glass showed and least solubility (1.07%). Meanwhile, GIC reinforced with 10 wt% showed intermediate results (3.2%), (*P* = 0.0001*).

**Table 8:** The solubility mean and standard deviation values among the groups (%)

Test	GIC (control)	GIC reinforced with 10 wt% E-glass		GIC reinforced with 20 wt% E-glass	*P* value
Solubility (%)	5.5^c^ ± 0.3		3.2^b^ ± 0.2	1.07^a^ ± 0.05	0.00001*

Different small letters in the same row are significant difference, * denotes significant difference as *P* ≤ 0.05

## Discussion

Traditional GICs are used widely in dentistry because of their distinctive characteristics, including their chemical adhesion to tooth structures, which require minimal dental preparation, the ability to release fluoride, biological computability, and thermal computability with enamel, all of which reduce the amount of tooth preparation needed [46]. However, one of the primary challenges with GICs is their inferior mechanical features [47]. Since most mastication forces are compressive, a compressive strength test resembles the load applied to materials used in dental treatment [48]. Moreover, the compressive strength of GIC is commonly measured after 24 h wet storage [49]. Both compressive and flexural analysis were employed to mimic the stress placed on the materials used in clinical dentistry.

The durability of the restorations greatly depends on the resistance of the restorative material to intraoral circumstances. When dental materials are exposed to the oral environment for extended periods of time, the contact may cause the surface layers to dissolve or deteriorate [50]. Solubility and surface hardness are important features that determine the longevity of the GIC [51]. Furthermore, the material’s clinical durability is significantly influenced by its flexural strength [22]. The ideal’ dental cement and restoration should have several features, such as high surface and mechanical characteristics, adequate setting time, and a low film thickness (less than 25 μm) for the luting agent [52].

There have been several attempts to improve the mechanical properties of the GICs by incorporating reinforcement filler [53]. High aspect ratio E-glass fibers reinforcement is used in dentistry as well as numerous other technical fields. However, they haven’t been thoroughly investigated with GICs [3].

The adhesion between the fibers and matrix has an important influence on the mechanical properties of the material [54]. To ensure that the load gets transferred to the stronger fibers, adequate adhesion between the fiber and matrix is necessary to ensure proper load transfer [54]. Therefore, the selected E-glass fibers were silane-treated in order to improve the adhesion of the fillers [33, 34].

The current study used two concentrations of E-glass fibers: 10 wt% and 20 wt%. According to a previous investigation, the strength of the restoration was reduced when fibers loading exceeded 25 wt% [54], which may be attributed to the fact that excessive fiber loading may create microstructural voids or flaws [54]. These microstructural flaws may have a determinate effect on the compressive strength and surface microhardness. Moreover, it may increase the solubility of the GIC [55].

This study was done to evaluate the effects of incorporating (10 wt% and 20 wt%) silane-treated E-glass fibers into conventional GIC on their compressive strength, flexural strength, hardness, and solubility. The results indicated that incorporation of E-glass fibers would enhance the mechanical performance of conventional GIC with an increase in compressive strength, flexural strength, surface hardness, and reduction in solubility compared to conventional GIC. The incorporation of a concentration of 20 wt% E-glass fibers provides a more favorable result than that of 10 wt%. Therefore, the null hypothesis of this study was rejected.

The chemical composition of the fibers was confirmed by X-ray spectrometry. The XRD data showed that the primary constituents of the E-glass fibers were SiO_2_, Al_2_O_3_, MgO, and CaO, with small amounts of B_2_O_3 and_ K_2_O. The results were consistent with the chemical content of the frequently utilized E-glass reinforcing fibers [56].

SEM imaging is an ideal method for analyzing the uniformity and homogeneity of glass fiber distribution [57]. The distribution, orientation, aspect ratio, and morphology of the fibers could be identified by the SEM examination [58]. The SEM image results of the reinforced fibers show a short and thin fiber structure which may be crucial for the possible strengthening effects. Moreover, the SEM image results after mixing the reinforced groups showed a nearly uniform distribution of a short E-glass fibers within the GIC, which denotes good incorporation of the E-glass fibers during the mixing procedure. Furthermore, there seems to be a tight bond between the E-glass fibers and the GIC, which may be attributed to the silane treatment of the E-glass fibers [33, 34], This indicates that the addition of E-glass fiber increases the GIC’s strength. On the other hand, some cracks exhibited on the surface of GIC may be due to dehydration during specimen preparation [11, 59]. The cracks noticed on the matrix surface may also be caused by the attack of freeze-dried acid polymers on the basic glass powder [60].

Particle size and distribution analysis are precise and essential techniques for enhancing the use of filler particles. It is believed to provide an accurate method for measuring the size of the particles [61]. Combining the diameter, length, and aspect ratio distribution data from the particle size analysis indicate a short glass fiber with a high aspect ratio, which may be crucial factors in determining the reinforcement potential of E-glass fibers [62].

Dental cement should have a film thickness of no more than 25 μm for water-based luting cements, in compliance with ADA No. 8 [63]. Enhanced marginal adaptation and improved restoration retention are the results of minimal film thickness of the dental cements [64]. Physical characterization results showed a film thickness of less than 25 μm is provided by both the treated and control groups with no significant difference between the them. Regarding the initial setting time, there was also no significant difference between the groups. These findings may be explained by the lower concentration of the incorporated short fibers.

The results of the compressive strength investigation showed that the modified groups, through the incorporation of E-glass fiber fillers into conventional GIC, significantly improved the compressive strength compared to the unmodified groups. This result could be explained by the predicted strengthening effect of the fillers made of E-glass fibers [32, 65]. The reinforcement effect may be due to the high rigidity of the E-glass fibers, which act as a crack stopper that prevents cracks from starting and spreading [3]. As a result, the material gains increased fracture resistance [66].

The improvement of the compressive strength in the group modified by the incorporation of 20 wt% E-glass fibers was more pronounced than that modified by the incorporation of only 10 wt% E-glass fibers. This may be attributed to the higher concentration of fibers, which provide a stronger effect [3, 67]. These findings come in agreement with the study provided by Sari et al. [3].

All of the reinforced groups in this study show an increase in flexural strength rather than the control group; this may be due to the adequate impregnation and bonding of silane-treated fibers to the GIC, which may prevent crack propagation by exerting a force opposing the crack [68]. Moreover, the maximum flexural strength was obtained by impregnation of E-glass fiber with GIC having a concentration of 20 wt%; this may be attributed to the increase in the reinforcing effect of the impregnated fibers [69].

The results of the surface hardness examination showed that the GIC reinforced with 20 wt% E-glass showed the highest hardness. While GIC reinforced with 10 wt% showed intermediate results, the unmodified GIC showed the least hardness. These results could be explained by the hard E-glass fiber filler phase that exists within the matrix and acts as the strongest reinforcement [70, 71].

In the present study, the GIC modified with 10 wt% and 20 wt% E-glass fiber addition reduced the solubility more than the unmodified GIC. The low solubility of the integrated E-glass fibers could potentially explain this finding [72, 73].

Other studies were conducted to improve the mechanical properties of the GIC. Murugan et al. [74], aim to improve the mechanical properties of the GIC by incorporating nanohydroxyapatite. They found that the reinforced GIC displayed increased compressive strength and fracture toughness and decreased cytotoxicity and microleakage. Beketova et al. [75], examined the effects of adding zirconia nano-fillers to GIC. They found that such an addition significantly improved the flexural strength and bond strength, decreasing water sorption without negatively affecting the film thickness. Moreover, Abed et al. [76], revealed that the addition of 4 wt% silver nanoparticles preserves the same bond quality as GIC while improving the mechanical characteristics. Furthermore, Chaudhary et al. [77]. Evaluated the effect of the addition of 3 wt% titanium dioxide nano powder and 10 wt% nanohydroxyapatite to GIC. The results showed that the GIC modified with titanium dioxide showed the highest flexural and compressive strength.

One of the study’s limitations is that the experimental conditions weren’t exactly like the clinical ones. Moreover, the release of the fluoride ions was not examined after the addition of E-glass fibers. Additionally, the study investigated the mechanical properties in only short-term storage time. It is suggested that more research be done to examine the potential impacts of adding E-glass fibers to GIC at varying aspect ratios, fiber orientations, and amounts. Moreover, it is recommended to do a further study to assess the surface roughness, Further studies are recommended to investigate the mechanical properties after prolonged soaking. Moreover, it is recommended to examine the remineralization potential after the incorporation of E-glass fibers.

## Conclusions

Compared to traditional GIC dental cement, the innovatively reinforced GIC with 20 wt% silane-treated E-glass fiber fillers offer improved compressive strength, flexural strength, hardness, and decreased solubility. As a result, it may be utilized as an alternative.

## Data Availability

The data that support the findings of this study are available from the corresponding author upon reasonable request.

## Abbreviations

GICs: Glass ionomer cements
wt.%: Weight%
MREC: Medical Research Ethical Committee
NRC: National Research Centre
XRF: X-ray fluorescence
SEM: Scanning electron microscope
PSD: Particle size distribution
DLS: Dynamic light scattering
ISO: International Organization for Standardization
MPa: Megapascal
VH: Vickers microhardness
VHN: Vickers hardness numbers
